# Supplementary material for: Social-affective features drive human representations of observed actions
Source: eLife. 2022 May 24;11:e75027. doi: 10.7554/eLife.75027 (PMC9159752; doi:10.7554/eLife.75027)
Supplement: Supplementary file 1. — (a) Breakdown of scene setting and number of agents across the two final stimulus sets. (b) Features quantified in both stimulus sets and used to generate feature representational dissimilarity matrices (RDMs) in the representational similarity analysis. [file elife-75027-supp1.docx]

1. Breakdown of scene setting and number of agents across the two final stimulus sets.

|  | Scene setting | | | Number of agents | | | |
| --- | --- | --- | --- | --- | --- | --- | --- |
|  | Indoors | Outdoors | Unclear | 0 | 1 | 2 | 3+ |
| Video set 1 | 89 | 60 | 3 | 8 | 52 | 50 | 42 |
| Video set 2 | 39 | 26 | 0 | 0 | 21 | 29 | 15 |

1. Features quantified in both stimulus sets and used to generate feature RDMs in the representational similarity analysis. *This feature was generated as a binary RDM clustering videos by category.

| Feature name | Feature type | Computation method | Numeric representation  (per video) |
| --- | --- | --- | --- |
| Pixel value | Visual | Automatic extraction | Real-valued vector (480,000D) |
| Hue | Visual | Automatic extraction | Real-valued vector (480,000D) |
| Saturation | Visual | Automatic extraction | Real-valued vector (480,000D) |
| Watermark | Visual | Experimenter labels | Value (0-1) |
| Gist | Visual | Automatic extraction | Real-valued vector (512D) |
| Environment | Visual | Experimenter labels | Value (0-1) |
| Optic flow | Visual | Automatic extraction | Real-valued vector (480,000D) |
| AlexNet Conv1 | Visual | Automatic extraction | Real-valued vector (69,984D) |
| AlexNet FC8 | Visual | Automatic extraction | Real-valued vector (1000D) |
| Action category | Action | Experimenter labels | Binary RDM* |
| Effectors | Action | Experimenter labels | Binary vector (5D) |
| Transitivity | Action | Behavioral ratings | Value (1-5) |
| Activity | Action | Behavioral ratings | Value (1-5) |
| Valence | Social-affective | Behavioral ratings | Value (1-5) |
| Arousal | Social-affective | Behavioral ratings | Value (1-5) |
| Sociality | Social-affective | Behavioral ratings | Value (1-5) |
| Number of agents | Social-affective | Experimenter labels | Value (0-3) |
