# Supplementary material for: Social-affective features drive human representations of observed actions
Source: eLife. 2022 May 24;11:e75027. doi: 10.7554/eLife.75027 (PMC9159752; doi:10.7554/eLife.75027)
Supplement: Transparent reporting form [file elife-75027-transrepform1.docx]

***eLife’s* transparent reporting form**

We encourage authors to provide detailed information *within their submission* to facilitate the interpretation and replication of experiments. Authors can upload supporting documentation to indicate the use of appropriate reporting guidelines for health-related research (see [EQUATOR Network](http://www.equator-network.org/%20)), life science research (see the [BioSharing Information Resource](https://biosharing.org/)), or the [ARRIVE guidelines](http://www.plosbiology.org/article/info:doi/10.1371/journal.pbio.1000412) for reporting work involving animal research. Where applicable, authors should refer to any relevant reporting standards documents in this form.

If you have any questions, please consult our Journal Policies and/or contact us: [editorial@elifesciences.org](mailto:editorial@elifesciences.org).

**Sample-size estimation**

- You should state whether an appropriate sample size was computed when the study was being designed
- You should state the statistical method of sample size computation and any required assumptions
- If no explicit power analysis was used, you should describe how you decided what sample (replicate) size (number) to use

Please outline where this information can be found within the submission (e.g., sections or figure legends), or explain why this information doesn’t apply to your submission:

For the multiple arrangement experiments, we conducted a pilot experiment with 20 participants and used this to determine a sufficient number of measurements per pair. Experiment 1 involved a large-scale stimulus set, and we achieved an average of 11.4 distance estimates for each of the 11,476 stimulus pairs with a sample size of N=300 (after exclusions). To replicate Experiment 1, distances between 2080 stimulus pairs were provided in Experiment 2 by N=53 participants (after exclusions), a sample size more than twice as large as that used in previous multiple arrangement studies.

Although we did not conduct a formal power analysis for the EEG experiment, the sample size (N=15) was based on comparable EEG studies showing reproducible effects with similar sample sizes and analyses (e.g. Greene & Hansen, 2020), as well as funding and time constraints, which were exacerbated by the pandemic. All details about sampling and participants are described in the Behavior: Participants and EEG: Participants sections.

**Replicates**

- You should report how often each experiment was performed
- You should include a definition of biological versus technical replication
- The data obtained should be provided and sufficient information should be provided to indicate the number of independent biological and/or technical replicates
- If you encountered any outliers, you should describe how these were handled
- Criteria for exclusion/inclusion of data should be clearly stated
- High-throughput sequence data should be uploaded before submission, with a private link for reviewers provided (these are available from both GEO and ArrayExpress)

Please outline where this information can be found within the submission (e.g., sections or figure legends), or explain why this information doesn’t apply to your submission:

The multiple arrangement experiment was replicated with a different stimulus set and participant pool. Participants in the behavioral experiments were excluded if they responded incorrectly to an action labeling question, and if the multiple arrangement they performed on a training set of 7 videos correlated poorly with other participants’ data. All exclusion criteria are described in detail in the Multiple arrangement section of the Methods.

The EEG experiment was conducted once, and no participants were excluded (see EEG: Participants section).

**Statistical reporting**

- Statistical analysis methods should be described and justified
- Raw data should be presented in figures whenever informative to do so (typically when N per group is less than 10)
- For each experiment, you should identify the statistical tests used, exact values of N, definitions of center, methods of multiple test correction, and dispersion and precision measures (e.g., mean, median, SD, SEM, confidence intervals; and, for the major substantive results, a measure of effect size (e.g., Pearson's r, Cohen's d)
- Report exact p-values wherever possible alongside the summary statistics and 95% confidence intervals. These should be reported for all key questions and not only when the p-value is less than 0.05.

Please outline where this information can be found within the submission (e.g., sections or figure legends), or explain why this information doesn’t apply to your submission:

A detailed presentation of the statistical tests used is provided in the Methods section (Behavior: Data analysis, EEG: Data analysis), as well as in figure captions.

(For large datasets, or papers with a very large number of statistical tests, you may upload a single table file with tests, Ns, etc., with reference to sections in the manuscript.)

**Group allocation**

- Indicate how samples were allocated into experimental groups (in the case of clinical studies, please specify allocation to treatment method); if randomization was used, please also state if restricted randomization was applied
- Indicate if masking was used during group allocation, data collection and/or data analysis

Please outline where this information can be found within the submission (e.g., sections or figure legends), or explain why this information doesn’t apply to your submission:

Participants were not allocated to different experimental groups. In behavioral Experiment 1, participants arranged different sets of stimuli, which were randomly drawn from the full stimulus set using an algorithm that ensured all stimuli were presented equally often across participants.

**Additional data files (“source data”)**

- We encourage you to upload relevant additional data files, such as numerical data that are represented as a graph in a figure, or as a summary table
- Where provided, these should be in the most useful format, and they can be uploaded as “Source data” files linked to a main figure or table
- Include model definition files including the full list of parameters used
- Include code used for data analysis (e.g., R, MatLab)
- Avoid stating that data files are “available upon request”

Please indicate the figures or tables for which source data files have been provided:

All data, including source data and the relevant code for generating Figures 1, 3, 4 and 5, are available on OSF: <https://osf.io/hrmxn/>.
